# An Evaluation of the Drug Permeability Properties of Human Cadaveric In Situ Tympanic and Round Window Membranes

**DOI:** 10.3390/ph15091037

**Published:** 2022-08-23

**Authors:** Joachim G. S. Veit, Bhaskar Birru, Yong Wang, Ruby Singh, Elizabeth M. Arrigali, Ryan Park, Briggs Miller, Matthew A. Firpo, Albert H. Park, Monica A. Serban

**Affiliations:** 1 Department of Biomedical and Pharmaceutical Sciences, University of Montana, Missoula, MT 59812, USA; 2 Montana Biotechnology Center (BIOTECH), University of Montana, Missoula, MT 59812, USA; 3 Department of Surgery, University of Utah School of Medicine, Salt Lake City, UT 84132, USA

**Keywords:** temporal bone, tympanic membrane, round window membrane, drug diffusion

## Abstract

It is estimated that hearing loss currently affects more than 1.5 billion people, or approximately 20% of the global population; however, presently, there are no Food and Drug Administration-approved therapeutics or prophylactics for this condition. While continued research on the development of otoprotective drugs to target this clear unmet need is an obvious path, there are numerous challenges to translating promising therapeutic candidates into human clinical testing. The screening of promising drug candidates relies exclusively on preclinical models. Current models do not permit the rapid high-throughput screening of promising drug candidates, and their relevance to clinical scenarios is often ambiguous. With the current study, we seek to understand the drug permeability properties of the cadaveric tympanic and round window membranes with the goal of generating knowledge that could inform the design and/or evaluation of in vitro organotypic models. The development of such models could enable the early high-throughput screening of topical therapeutic candidates and should address some of the limitations of currently used animal models.

## 1. Introduction

Hearing loss (HL) is a debilitating condition affecting approximately 20% of the global population [1]. It is projected that by 2050, one in ten people will suffer from HL with direct effects on their quality of life [2]. In addition to the negative impacts on many aspects of the lives of those affected, in 2019 it was estimated that the total global economic costs of hearing loss exceeded USD 981 billion [3].

These statistical data compellingly highlight the need for effective preventive or therapeutic strategies against HL, yet, to date, there are no Food and Drug Administration-approved drugs for this condition [4]. The research focusing on identifying effective prophylactics or therapeutics is ongoing; however, there are numerous hurdles in successfully advancing promising drug candidates to clinically available products. The task is particularly challenging for therapeutics addressing sensorineural hearing loss (SNHL), where the affected internal, cochlear structures of the ear and the neural pathways to the auditory cortex are difficult to access [5,6,7]. In such cases, the challenge is three-fold as it requires the identification of a safe and effective drug, a performant drug formulation, and an efficient drug delivery system that prioritizes patient comfort [8].

Oto-therapeutics can be delivered to inner ear structures either systemically or topically. Systemic drug delivery to the cochlear structures would be an obvious non-invasive strategy for HL drug development; however, inner ear tissues are not easily accessible to blood-borne drugs due to the barrier between the vasculature and inner ear fluids that bathe hearing sensory cells (the blood–labyrinth barriers or BLBs) [9,10]. In addition, some agents, when administered systemically, may cause collateral adverse effects to susceptible organ systems. With the existing limited understanding of the fundamental parameters that dictate BLB permeation properties for individual systemically administered drugs [8], the development of drug formulations and optimization of delivery systems are especially challenging.

Consequently, the research on local therapeutic delivery routes into the inner ear has expanded in recent years [11]; however, the feasibility of topical delivery approaches are dictated by the anatomy of the ear [12]. To achieve non-invasive, topical access to the inner ear structures damaged in SNHL, an ideal therapeutic would have to be applied into the outer ear canal, permeate through the tympanic membrane (TM), diffuse through the middle ear structures, and then reach and permeate the oval and/or round window membrane (RWM). Subsequently, the drug would need to reach the affected cochlear or neural structures at a concentration suitable for therapeutic action. 

Given the innate barrier properties of the TM, a first step in considering a non-invasive topical delivery approach would be to comprehensively consider the amount of drug able to permeate the TM; the impact of the TM structure on drug flux; and the intrinsic drug properties interacting with the membrane with respect to their adsorption, distribution, metabolism, and elimination [13]. Problematically, this initial TM permeation has been associated with an over 100-fold decrease in drug concentration [14], prompting researchers to consider delivery methodologies that physically circumvent the TM, such as transtympanic injections (often referred to as intratympanic) directly into the middle ear [15]. Other drug delivery methodologies that explored deployment directly onto the RWM, or into the vestibular structures, using various delivery devices have been reported; however, these techniques are invasive [16,17,18]. An additional challenge in the development of effective topically delivered oto-therapeutics is the accurate detection and quantification of the drug amount that reaches the targeted inner ear structures [8,16]. Consequently, the direct assessment of drug delivery into the inner ear structures has only been performed in a third of the studies focusing on topical drug delivery, with the majority of the studies assessing therapeutic efficacy via clinical outcomes [11]. While the challenges associated with the development of safe and effective topical oto-therapeutics seem overwhelming, the advantages of increasing therapeutic efficacy at the target site while eliminating the adverse effects associated with systemic delivery continues to drive research efforts in this area.

From a research and development perspective, decoupling the evaluation of a potential drug candidate from the drug delivery system can be difficult. Deconstructed, simple, yet physiologically relevant, model systems that could add to our current understanding of the intrinsic drug properties and individual TM and RWM drug-permeation parameters that drive the prophylactic or therapeutic outcomes of topically delivered drugs would be a valuable tool for the rapid, high-throughput screening of novel drug candidates against HL.

This study is intended to serve as a first step in the development of deconstructed in vitro models and focuses on the assessment of the drug permeation parameters of human cadaveric TM and RWM for two clinically relevant drugs: dexamethasone sodium phosphate (DSP) and ciprofloxacin hydrocholoride (HCl). The results obtained from this study build on previously reported human cadaveric TM studies that assessed the concentration-dependent permeation of ciprofloxacin [19], and additionally compares the membrane permeation properties of two drugs with different partition coefficients (log P) in both TM and RWM. 

## 2. Results

### 2.1. Isolation of In Situ TMs and RWMs 

**Figure 1:**
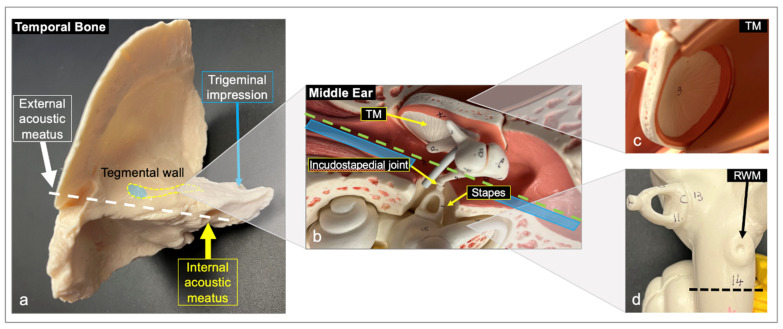
Representation of the temporal bone processing technique on anatomical models. (**a**) Model of human TB. The dotted, white line indicates the plane between the external acoustic meatus (ear canal) and the internal acoustic meatus; the area marked by a dotted, yellow line represents the drill cuts used to access the middle ear structures; the blue oval area represents the approximate position where the middle ear would be exposed. (**b**) Model of exposed middle ear. Following cleavage of the incudostapedial joint, the TB was split along the dotted, green line to separate the TM and RWM. (**c**) Model of the TM held within the bone as observed from a partial cutaway of the external acoustic meatus. (**d**) Model of the inner ear labyrinth containing the RWM. The dotted, black line represents the cut created to access the backside of the RWM for diffused drug collection. RWM, round window membrane; TB, temporal bone; TM, tympanic membrane.

To isolate in situ TMs and RWMs, the middle ear was accessed via the removal of tegmen tympani using an otologic drill (Figure 1). This maneuver provided excellent exposure of the middle ear structures (Figure 1a). Once the middle ear structures were exposed, the incudostapedial joint connecting the incus and the stapes was gently cleaved with surgical scissors to allow for the independent separation of the planes containing the TM and RWM (Figure 1b: dotted, green line). Cuts were then extended anteriorly and posteriorly (Figure 1b: blue areas) to separate the stapes and round window from the tympanic membrane. 

**Figure 2:**
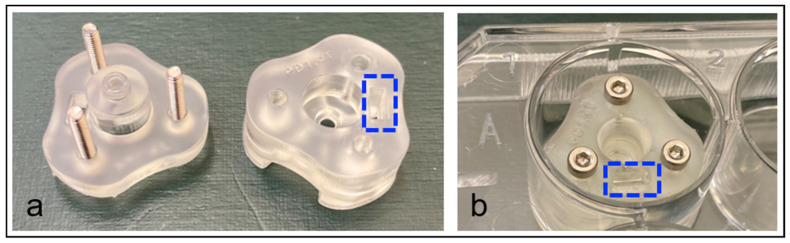
Custom drug permeation devices used for the evaluation of biopsied TM samples. (**a**) Photograph of the 3D-printed permeation device used to affix a 3 mm diameter biopsy of cadaveric TM for drug permeation studies; the tissue sample is positioned in the 3 mm diameter cavity in the base (**right**), then covered with the device top (**left**), and secured in place with bolts and embedded nuts; the device contains a slot (blue, dotted rectangle) for electrode access to the underside to allow for the assessment of the quality of the tissue seal via TEER measurements. (**b**) The assembled permeation device is placed in a 12-well tissue culture plate and a drug solution was placed in the upper donor chamber (central channel), while the permeated drug is collected from the well below. TM, tympanic membrane; TEER, transepithelial electrical resistance.

To completely separate the two tissue planes (one containing the TM and the other one the RWM structures), additional cuts were made to incorporate the inner ear (medial cuts) and a portion of the ear canal (lateral cuts). The two planes containing the intact in situ TM and RWM (identified using the stapes as landmark) were then gently separated. The TM and RWM containing bones were trimmed further to approximately 5 × 5 cm (Figure 1c,d: dotted, black line). To enable the collection of the drugs permeating the RWM, a final cut was made in the cochlea adjacent to the RWM and perpendicular to the surface to gain access to the backside of the RWM (Figure 1d: dotted, black line). Given the complexity of the isolation procedure, the integrity of several TMs was compromised with membranes partially rupturing during the isolation process. For those samples, when allowed by the integrity of the recovered tissue, the TM was excised along the annulus using a fine-tipped scalpel and placed into phosphate-buffered saline (PBS). A 3 mm biopsy punch was used on the intact portion of excised TM, and the biopsied tissue was mounted into a custom 3D-printed permeation device (Figure 2). These samples were evaluated for their drug permeability properties alongside the in in samples. 

### 2.2. Evaluation of TM Barrier Properties

**Figure 3:**
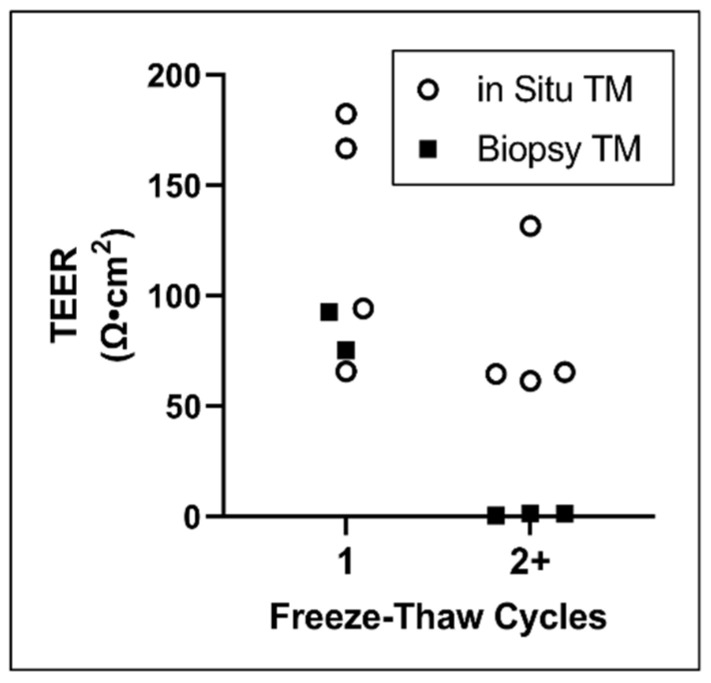
Impact of freeze–thaw cycles on TM barrier properties. TEER values of TMs from TBs frozen/thawed once or multiple times. Lines show mean. TEER, transepithelial electrical resistance; TM, tympanic membrane.

The barrier integrity of TM samples that underwent single or multiple freeze/thaw cycles was evaluated via transepithelial electrical resistance (TEER). In situ and biopsied tissues mounted in permeation devices were collectively evaluated (Figure 3). As previously reported [19], the data show that subjecting tissues to multiple freeze/thaw cycles compromises TM barrier integrity.

### 2.3. Drug Permeation in TM

**Figure 4:**
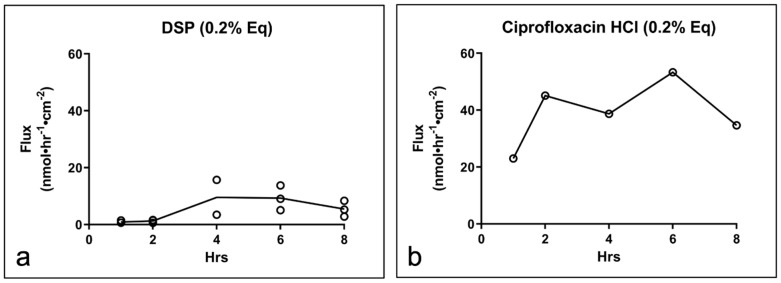
Drug permeation across in situ TMs isolated from once-frozen TB. Drug flux of (**a**) DSP (*n* = 3) and (**b**) ciprofloxacin HCl (*n* = 1) through in situ TM. TM, tympanic membrane; TB, temporal bone; DSP, dexamethasone sodium phosphate; Eq, molar equivalent of base drug.

The permeation of DSP and ciprofloxacin HCl was assessed in intact, in situ TMs isolated from once-frozen TBs over 8 h (Figure 4). For DSP (*n* = 3), the drug flux started to approach a steady state (SS) following approximately 4 h (flux_ss_ = 8.1 nmol·hr^−1^·cm^−2^, P_app_ = 1.60 × 10^−3^ cm·hr^−1^) (Figure 4a), while ciprofloxacin HCl (*n* = 1) appeared to reach SS following 2 h (flux_ss_ = 43.0 nmol·hr^−1^·cm^−2^, P_app_ = 7.11 × 10^−3^ cm·hr^−1^) (Figure 4b).

**Figure 5:**
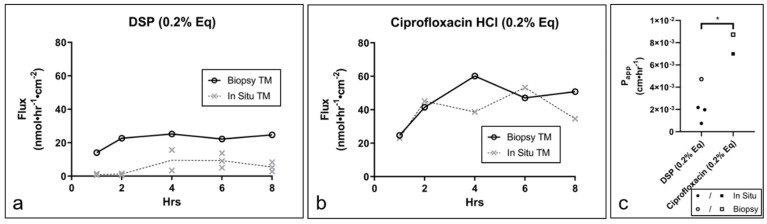
Drug permeation in cadaveric TM biopsies fixed in permeation devices. Drug flux of (**a**) DSP (*n* = 1) and (**b**) ciprofloxacin HCl (*n* = 1) in TM biopsies relative to flux in in situ TM (dotted, white line). Lines show mean. (**c**) Apparent permeability coefficients (P_app_) of DSP (*n* = 4) and ciprofloxacin HCl (*n* = 2) for combined in situ and biopsied TM. * *p* < 0.05; *t*-test. TM, tympanic membrane; DSP, dexamethasone sodium phosphate; Eq, molar equivalent of base drug; P_app_, apparent permeability coefficient.

**Figure 6:**
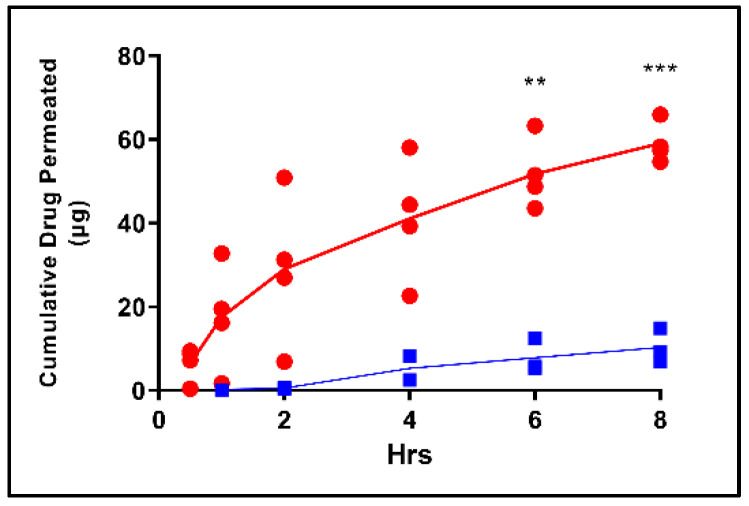
Impact of freeze–thaw cycles on TM drug permeation. Cumulative DSP permeated across in situ TMs, which underwent one (blue, *n* = 3) or multiple (red, *n* = 4) freeze–thaw cycles. Lines show mean. ** *p* < 0.01, *** *p* < 0.001; multiple *t*-tests (per timepoint) with Holm–Šídák correction for multiple comparisons. TM, tympanic membrane; DSP, dexamethasone sodium phosphate.

Two additional biopsied TM tissues were also tested for drug permeability (DSP and ciprofloxacin HCl, *n* = 1 each) with flux trends comparable to those observed in intact, in situ tissues (Figure 5a,b). A comparison of the P_app_ values for each drug in combined in situ and biopsied samples (*n* = 4 for DSP and *n* = 2 for ciprofloxacin HCl) indicated that the permeability of DSP was significantly lower than that of ciprofloxacin HCl (Figure 5c). As reflected by TEER, the number of tissue freeze/thaw cycles affected TM barrier properties of the TM, which translated to significantly increased drug permeation activity (Figure 6). 

### 2.4. Drug Permeation in RWM

**Figure 7:**
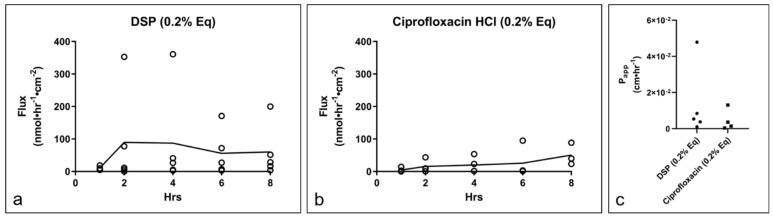
Drug permeation across in situ RWM. Flux of (**a**) DSP (*n* = 5) and (**b**) ciprofloxacin HCl (*n* = 4) across in situ RWM. (**c**) P_app_ of each drug in RWM. Lines show mean. RWM, round window membrane; DSP, dexamethasone sodium phosphate; Eq, molar equivalent of base drug; P_app_, apparent permeability coefficient.

Subsequently, the drug permeation properties of DSP and ciprofloxacin HCl in RWM were assessed over 8 h. In in situ RWMs, the DSP flux appeared to reach SS by hour 2 (Figure 7a) (flux_ss_ = 73.2 nmol·hr^−1^·cm^−2^, P_app_ = 1.44 × 10^−2^ cm·hr^−1^), while ciprofloxacin HCl (Figure 7b) appeared to reach SS at hour 2 (flux_ss_= 50.5 nmol·hr^−1^·cm^−2^, P_app_ = 8.37 × 10^−3^ cm·hr^−1^). No statistically significant differences were observed between the flux or permeability coefficient (Figure 7c) for DSP and ciprofloxacin HCl in RWMs. 

## 3. Discussion

As indicated in the introduction, this study intended to serve as a first step in the development of deconstructed in vitro models of TM and RWM to enable the rapid, high-throughput screening of novel drug candidates against hearing loss. Herein, a novel method for the isolation of in situ TM and RWM was introduced, an alternative for maximizing the use of non-intact TMs via 3D-printed drug permeation devices was described, and the drug permeation properties of two clinically relevant drugs were assessed.

The diffusion experiments in this study were conducted at room temperature to decrease the rate of tissue degradation, yet preserve physiological relevance, and the humidity of the tissues during the experiments was maintained by keeping the plates enclosed in plastic bags containing moist paper towels. No tissue desiccation was observed during our experiments. The duration of the drug permeation experiments was limited to 8 h, which appeared sufficient to achieve a steady state in all cases and minimized the effect of natural tissue degradation. 

The drug permeation experiments were conducted with a constant concentration gradient under sink conditions, and the flux of the tested drugs, expressed as the number of molecules moving through the cross-sectional area of a membrane in a given period of time, was determined [20]. The two drugs included in this study were selected based on their clinical relevance to otic pathologies and were used at concentrations reflective of those in commercially available dosage forms (Decadron, Cetrexal, Cipro HC) [21,22,23,24,25,26]. Additionally, based on their partition coefficients, DSP was more lipophilic (log P = 1.56) [27] compared to the more hydrophilic ciprofloxacin HCl (log P = −0.86) [28]. 

Starting at the outer ear canal, the TM consists of an outer layer of stratified squamous keratinized epithelium, a middle fibro-elastic connective tissue layer and an inner layer comprised of a cuboidal mucosal epithelium [12,13]. Considering the lipid-rich stratified squamous keratinized epithelium of the TM and its previously reported affinity for small, moderately lipophilic molecules, a predominantly transcellular drug diffusion mechanism would be anticipated for the two drugs [12,29]. We believed that the differences in the flux values observed for the two drugs could be a reflection of their inherent permeability, which is largely dictated by log P and molecular weight [13], with the larger and more lipophilic DSP becoming sequestered more readily in the tissue. Interestingly, our biopsied samples generated a drug flux comparable to the intact, in situ TMs. The DSP samples showed somewhat lower permeability values, which could also be explained by increased sequestration by the tissue surrounding in situ TM; it could also be a reflection of the heterogeneous composition of the TM, which may be more apparent in biopsied samples than in intact, whole membranes [30,31,32].

It is important to note that ciprofloxacin permeation reported in a recent study conducted in fresh, never-frozen tissues [19] was approximately 40-fold lower than the permeation detected in our frozen samples. The study [19] also reported a TM barrier impairment in response to freeze/thaw cycles consistent with our own observations, which translated to more permeable membranes. Additionally, our data showed that the permeability of DSP was significantly higher in samples undergoing multiple freeze/thaw cycles than those experiencing only a single freeze/thaw cycle. 

In RWMs, the flux of the two drugs was not statistically different. Relative to the TM, the RWM seems to have a higher permeability for DSP (RWM P_app_ = 1.44 × 10^−2^ cm·hr^−1^ versus TM P_app_ = 1.60 × 10^−3^ cm·hr^−1^) and comparable permeability for ciprofloxacin HCl (RWM P_app_ = 8.37 × 10^−3^ cm·hr^−1^ versus P_app_ = 7.11 × 10^−3^ cm·hr^−1^). Unlike the stratified epidermis of the TM, the outer layer of the RWM consists of a single cell layer [33], more permeable [12], outer epithelium, which may explain the observed lack of definition between the permeability of the two drugs. The RWM additionally consists of a fibroblast-containing a connective tissue middle layer and a layer of squamous cells towards the inner ear [33]. Previous reports suggest that the outer epithelial layer of the RWM is the key determinant of the membrane’s permeation properties [34]. 

Overall, our study’s findings are well-aligned with the previously published data on ciprofloxacin permeation across the TM. We also assessed the permeability of an additional, more lipophilic, clinically relevant drug, DSP, and to our knowledge, for the first time determined the permeation properties of ciprofloxacin HCl and DSP in in situ human cadaveric RWMs. 

## 4. Materials and Methods

### 4.1. Temporal Bones

Ten once-frozen, fully de-identified temporal bones (TBs) were procured from Science Care (Phoenix, AZ). Seven fully de-identified human cadaveric TBs frozen/thawed at least two times were obtained from the Body Donor Program (University of Utah, Salt Lake City, UT). The use of de-identified cadaveric samples is not subject to ethical review and approval. In preparation for TM and RWM isolations, temporal bones were thawed overnight at 4 °C (for the frozen/thawed-at-least-two-times tissues) or packaged in plastic bags and under cold, running water (for the once-frozen tissues). Soft tissue was removed with scissors and surgical curettes, and the dura covering the superior aspect of the temporal bone was removed. Cerumen present in the external auditory canal was removed. Initial rough cuts were made with an autopsy saw, while fine cuts were made later with an otologic or piezoelectric drill fitted with carbide drill bits. A detailed description of the in situ tympanic membrane and round window membrane isolation procedure is included in the Results Section. In instances when the TM could not be isolated intact, 3 mm biopsy punches were used to recover intact tissue fragments that were subsequently analyzed for drug permeation properties, as described in the subsequent section. 

### 4.2. Permeation Device for TM Biopsies

A custom permeation device, as shown in the Results Section, was designed to allow for permeation testing to be performed on TM tissues in the absence of other tissue interactions, and if in situ testing had been compromised during TB processing. The permeation device was designed in Fusion360 software (Autodesk, San Rafael, CA, USA) and printed on a Form 3 (Formlabs, Somerville, MA, USA) stereolithographic 3D printer using Clear V4 (Formlabs) photopolymer resin. The device was designed to fit in a standard 12-well tissue culture plate, accept a 3 mm diameter circular tissue, and had a 2 mm diameter internal channel for permeation testing. An adjacent channel for electrode placement allowed for simplified TEER measurements to quantify tissue integrity and validate a proper seal was obtained during mounting. Following the tissue placement, the two printed parts were joined with three stainless-steel M2 bolts passing through printed channels and three nuts that had been press-fit into the base of the unit.

### 4.3. Transepithelial Electrical Resistance (TEER) and Surface Area Determination

TEER values were measured in biopsied and in situ TM samples prior to permeation testing with a Millicell ERS-2 voltohmmeter (Millipore, MERS00002). For in situ TM, the external acoustic meatus (EAM, ear canal) was filled with phosphate-buffered saline (PBS) and the middle ear side of the TM was submerged in PBS. One electrode probe was placed in the EAM and the other adjacent to the middle ear side of the TM. For biopsied TM, a 3 mm biopsy of TM was mounted in a permeation device, taking care to mount the external side of the TM facing up. The device was then placed in a 12-well tissue culture plate containing 1 mL of PBS, and 0.1 mL of PBS was placed into the upper donor chamber without trapping any air bubbles. One electrode was placed in the upper chamber, while the other was placed in the adjacent channel leading to the bottom of the well. Blank TEER measurements of the empty permeation device (biopsy) or electrodes in PBS (in situ) and the permeable surface area of the TMs were taken into account to allow the TM’s direct contribution to the TEER to be determined. 

A major and minor axis diameter of in situ TM was measured from the middle ear side of the TM using an Ultra Cal V caliper (Fowler, Canton, MA), and the surface area was approximated using the area of an ellipse: Surface area=Major radius∗Minor radius∗π. The surface area of the biopsied TM samples was defined using the permeation device’s circular channel diameter of 2 mm. In the RWM, an average surface area of 2.98 mm^2^ was used based on a previous literature report [35].

### 4.4. Drug Preparation

Dexamethasone 21-phosphate disodium (Alfa Aesar, J64083) was prepared at 2.63 mg·mL^−1^ (equivalent to 0.2% *w*/*v* dexamethasone) in PBS. Ciprofloxacin hydrochloride monohydrate (Alfa Aesar, J61970) was prepared at 2.33 mg·mL^−1^ (equivalent to 0.2% *w*/*v* ciprofloxacin) in PBS and the pH was adjusted to 3.5 ± 0.5 with 1 M hydrochloric acid to solubilize.

### 4.5. Drug Permeation Testing

Following the TEER measurement, in situ TMs were propped up with the EAM facing upwards in a 6-well tissue culture plate containing 750 µL of PBS (receiver solution), and 300 µL of drug solution was placed into the EAM (enough to cover the entire TM). At each timepoint (1, 2, 4, 6, and 8 h) the receiver solution was used to gently wash the middle ear side of the TM 4–5 times with a pipette before collection. To accurately determine the steady-state flux of the tissue, the concentration gradient of the donor and receiver solutions must remain constant throughout the testing. To avoid a noticeable decrease in the drug concentration, 150 µL of drug was removed from the EAM and replaced with fresh drug at each timepoint. The tissue was then moved to a new well containing fresh receiver solution until the next timepoint.

Following the TEER measurement, biopsied TMs mounted in permeation devices were placed in a 12-well tissue culture plate containing 750 µL of PBS (receiver solution), and 150 µL of drug solution was placed into the donor chamber, being sure that no air bubbles became trapped above or below the tissue. At each timepoint, the device was removed from the well, allowed to drip, then transferred to a fresh well containing fresh donor solution. To ensure a consistent donor-chamber concentration, 100 µL of drug solution was removed from the donor channel and replaced with fresh drug at each timepoint.

In situ RWMs were placed into a 6-well plate containing 750 µL of PBS (receiver solution) with the RWM facing up, and 5 µL of drug solution was placed directly into the round window niche. At each timepoint, the receiver solution was used to gently wash the backside of the RWM 4–5 times with a pipette, being careful not to allow the receiver or drug solutions to mix. The tissue was then moved to a new well containing fresh receiver solution. If the drug solution remained in the round window niche, it was gently wicked away with a lab tissue and replaced with 5 µL of fresh drug solution.

All receiver solutions were collected immediately in microfuge tubes and stored at −20 °C until analysis. Between timepoints, the tissues were placed in a plastic bag containing a moist paper towel to maintain humidity and protected from light under aluminum foil. Any noticeable decrease in donor solution level in in situ or biopsied TM indicated a leak or compromised tissue and the sample was excluded from the study.

### 4.6. High-Performance Liquid Chromatography

All samples were filtered with 0.22 µm PVDF syringe filters prior to HPLC analysis. All reagents used were HPLC grade; the buffers were freshly made and filtered at 0.1 µm prior to use. HPLC was performed using an Agilent 1260 Infinity II system (Agilent Technologies Inc., Santa Clara, CA, USA). 

For DSP detection, 50 µL of sample was injected into an isocratic mobile phase of 75% 0.01 M potassium phosphate buffer (pH 7.58) and 25% acetonitrile flowing at 1.5 mL·min^−1^ through a Gemini 3 µm 100 × 4.6 mm reverse-phase C_18_ column (Phenomenex, 00D-4439-E0) protected by a SecurityGuard C_18_ 4 × 3.0 mm cartridge (Phenomenex, AJ0-7597) at 25 °C. The retention time of DSP at 1.9 min was used to quantify the area under the curve (AUC) of absorbance at 240 nm. 

For ciprofloxacin HCl detection, 50 µL of sample was injected into an isocratic mobile phase of 80% 0.02 M potassium phosphate buffer (pH to 2.7 with orthophosphoric acid) and 20% acetonitrile flowing at 1.0 mL·min^−1^ through a Gemini 5 µm 150 × 3 mm reverse-phase C_18_ column (Phenomenex, 00F-4435-Y0) protected by a SecurityGuard C_18_ 4 × 2.0 mm cartridge (Phenomenex, AJ0-7596) at 25 °C. The retention time of ciprofloxacin HCl at 1.75 min was used to quantify the area under the curve (AUC) of absorbance at 277 nm.

### 4.7. Analysis of Flux and Permeability Coefficient

After determining the concentration of drug in the receiver solution by HPLC, the amount of drug in the total volume collected was calculated and the flux was calculated as follows: Flux=D ÷ ΔT ÷ A, where *D* is the nmol of drug permeated since the previous timepoint, Δ*T* is the time (hours) elapsed since the previous timepoint, and *A* is the permeable area (cm^2^) of the measured individual tested TM or the literature value of an average RWM [35].

Apparent permeability coefficients (P_app_, cm·hr^−1^) were calculated using Fick’s law, $\mathrm{Papp}=\frac{Flux}{C_{d}-C_{r}}$, where *C_d_* is the donor solution concentration (nmol·cm^−3^), which was considered constant throughout the experiment as excess was provided and replenished at each timepoint; *C_r_* is the receiver solution concentration, which was considered constant at 0 as the solution was replaced at each timepoint and any accumulation between timepoints remained negligible relative to *C_d_*, which was confirmed with HPLC.

### 4.8. Statistical Analysis

Prism version 9 software (GraphPad, San Diego, CA, USA) was used to perform the statistical analysis. The respective test used for each comparison was detailed in each corresponding figure.

## 5. Conclusions

This study aimed to assess the drug permeation parameters of the human cadaveric TM and RWM for two clinically relevant drugs, with the intent to serve as a first step in enabling the development of in vitro TM and RWM models that could allow the rapid, high-throughput screening of novel topically deliverable oto-therapeutic candidates. We described a novel method for the isolation of in situ human cadaveric TM and RWM, a method for assessing drug permeation in biopsied TM samples, and determined the flux of DSP and ciprofloxacin HCl in both TM and RWM. The limitations of using human cadaveric tissues for drug permeation studies have been previously well described [19]; however, the findings of this study could inform the development of physiologically representative in vitro TM and RWM models, which would help accelerate the discovery process for novel, locally deliverable, minimal, or non-invasive prophylactics and therapeutics for otic pathologies.

## Data Availability

Data is contained within the article.
